# Effect of Antimicrobial Peptide BiF2_5K7K on Contaminated Bacteria Isolated from Boar Semen and Semen Qualities during Preservation and Subsequent Fertility Test on Pig Farm

**DOI:** 10.3390/antibiotics13070579

**Published:** 2024-06-22

**Authors:** Krittika Keeratikunakorn, Panida Chanapiwat, Ratchaneewan Aunpad, Natharin Ngamwongsatit, Kampon Kaeoket

**Affiliations:** 1 Semen Laboratory, Department of Clinical Sciences and Public Health, Faculty of Veterinary Science, Mahidol University, 999 Phuttamonthon 4 Rd., Salaya, Phuttamonthon, Nakhon Pathom 73170, Thailand; krittika.ker@student.mahidol.edu (K.K.); panida.chn@mahidol.edu (P.C.); 2 Graduate Program in Biomedical Sciences, Faculty of Allied Health Sciences, Thammasat University, Rangsit Campus, Klong Luang, Pathumthani 12120, Thailand; aratchan@tu.ac.th; 3 Department of Clinical Sciences and Public Health, Faculty of Veterinary Science, Mahidol University, 999 Phuttamonthon 4 Rd., Salaya, Phuttamonthon, Nakhon Pathom 73170, Thailand; natharin.nga@mahidol.edu; 4 Laboratory of Bacteria, Veterinary Diagnostic Center, Faculty of Veterinary Science, Mahidol University, 999 Phuttamonthon 4 Rd., Salaya, Phuttamonthon, Nakhon Pathom 73170, Thailand

**Keywords:** antimicrobial peptide, boar semen, semen extender, semen quality

## Abstract

The purpose of this study was to determine the impact of an antimicrobial peptide, BiF2_5K7K, on semen quality and bacterial contamination in boar semen doses used for artificial insemination. A key factor affecting semen quality and farm production is bacterial contamination in semen doses. Using antibiotics in a semen extender seems to be the best solution for minimizing bacterial growth during semen preservation. However, concern regarding antibiotic-resistant microorganisms has grown globally. As a result, antimicrobial peptides have emerged as interesting alternative antimicrobial agents to replace the current antibiotics used in semen extenders. BiF2_5K7K is an antimicrobial peptide that can inhibit Gram-negative and Gram-positive bacteria isolated from boar semen and sow vaginal discharge. In this study, ten fresh boar semen samples were collected and diluted with one of two types of semen extender: with (positive control) or without (negative control) an antibiotic (i.e., gentamicin). The semen extender without an antibiotic contained antimicrobial peptide BiF2_5K7K at different concentrations (15.625, 31.25, 62.5, and 125 µg/mL). The samples were stored at 18 °C until use. Semen quality parameters were assessed on days 0, 1, 3, and 5, and the total bacterial count was also evaluated at 0, 24, 36, 48, and 72 h after storage. A fertility test on a pig farm was also performed via sow insemination with a commercial extender plus BiF2_5K7K at a concentration of 31.25 µg/mL. No significant difference was found in terms of semen quality on days 0 or 1. On days 3 and 5, the total motility, progressive motility, and viability remained normal in the 15.625 and 31.25 µg/mL groups. However, the sperm parameters decreased starting on day 3 for the 125 µg/mL group and on day 5 for the 62.5 µg/mL group. For total bacterial count at 0, 24, 36, 48, and 72 h, the lowest bacterial count was found in the positive control group, and the highest bacterial count was found in the negative control group compared with the other groups. Comparing antimicrobial peptide groups from 0 to 48 h, the lowest bacterial count was found in the 125 µg/mL group, and the highest bacterial count was found in the 15.625 µg/mL group. For the fertility test, artificial insemination was conducted by using a commercial extender plus BiF2_5K7K at a concentration of 31.25 µg/mL. The results showed a superior pregnancy rate, farrowing rate, and total number of piglets born compared with artificial insemination conducted using a commercial extender plus antibiotic. In conclusion, BiF2_5K7K can inhibit bacterial growth in extended boar semen for 24 h, and thereafter, the bacterial count slightly increases. However, the increase in the number of bacterial counts from days 0 to 3 had no negative effect on sperm quality in the positive control, 15.625, or 31.25 µg/mL groups. This indicates that BiF2_5K7K might be an antimicrobial peptide candidate with potential for use as an alternative antimicrobial agent to replace the conventional antibiotic used in boar semen extenders.

## 1. Introduction

Liquid boar semen preservation is routinely used in artificial insemination (AI) in the swine industry [1,2], as AI can reduce the risk of disease transmission and improve genetics, as well as increase the production or quality of piglets [1,3]. In the modern pig business, more than 93% of sows are inseminated via artificial insemination, and boar semen diluted with semen extenders is mostly used in the breeding herd [2]. The use of a semen extender is necessary to support the longevity and quality of sperm; that is, the extender protects sperm from cold shock, controls pH and osmotic pressure, and inhibits bacterial growth [1].

Although AI can reduce the transmission of disease from boar to gilt/sow, bacterial contamination in semen may affect their reproductive performance [3]. Bacteriospermia in humans and animals results in reduced quality, quantity, and longevity of spermatozoa [3,4]. In addition to reduced semen quality, it can result in embryonic or fetal death, endometritis, and vaginal discharge in sows [3]. Fresh boar sperm contain both Gram-negative and Gram-positive bacteria, including *Staphylococcus* spp., *Streptococcus* spp., *E*. *coli*, *Klebsiella* spp., *Aeromonas* spp., *Pseudomonas* spp., *Proteus* spp., and *Providencia* spp. [5,6,7,8]. It has been reported that contamination of boar semen with *E*. *coli*, *Proteus mirabilis*, *Pseudomonas aeruginosa*, *Clostridium perfringens*, and *Staphylococcus aureus* not only causes poor motility, but also reduces the integrity of the sperm membrane and acrosome [2]. Antibiotics play an important role in boar semen extenders, controlling bacterial contamination, reducing transmission of pathogens to the gilt/sow, and increasing the longevity of spermatozoa during storage [4,9].

It has been reported in tropical countries, including Thailand, that bacteria found in boar semen have developed resistance to multiple antibiotics commonly used in pig farms and added to boar semen extender. These antibiotics include amoxicillin, gentamicin, and colistin [8,10]. Consequently, bacteria isolated from boar semen have shown critical antibiotic resistance genes such as *mcr-3* and *int1* [8,10]. The medical world is concerned about antibiotic resistance, as many antibiotics are liberally used not only in humans, but also in livestock, and the pace of new antibiotic discoveries is slow [11]. In practice, many antibiotics are mixed into semen extenders to inhibit bacterial growth and limit the deleterious effects of contamination [4,9,12]. Gentamicin, neomycin, streptomycin, and other antibiotics are commonly used in boar semen extenders [13,14,15]. In many cases, more than one antibiotic is mixed into the boar semen extender; for example, combinations of gentamicin and florfenicol as well as gentamicin and polymyxin B have been used [16]. Since the emergence of antibiotic-resistant bacteria, many alternative antibacterial agents have been studied to reduce the use of antibiotics, including antimicrobial peptides (AMPs) [17]. To date, more than 2500 AMPs have been deposited in the Antimicrobial Peptide Database (APD) [18]. Antimicrobial peptides (AMPs), including proline-rich antimicrobial peptides (PrAMPs), tryptophan- and arginine-rich antimicrobial peptides, histidine-rich antimicrobial peptides, and glycine-rich antimicrobial peptides, have been identified as potential antimicrobial agents. These peptides show potential in combating antibiotic-resistant bacteria [19,20,21]. AMPs provide an alternative option to reduce or replace antibiotics used in swine and poultry production [22]. As observed since 2004, the number of publications on the topic of AMPs has increased every year [23]. In human medicine, AMPs, including Nisin A S26A, S29D, and S29E, have been applied for the prevention of food-borne pathogens such as *E*. *coli* and *Salmonella* Typhimurium [24]. In addition to using AMPs to prevent food-borne disease, AMPs are utilized as additional medical treatments, for example, in the treatment of burn wound infections using PXL150 and D2A21 [25]. With regard to the broad-spectrum antimicrobial activities of antimicrobial peptides, they are used to replace antibiotics in pig farms as growth promoters [26]. In a meta-analysis study, it was found that AMPs can improve average daily gain (ADG) and decrease the diarrhea rate in piglets [26]. The primary characteristic of AMPs is their ability to eliminate bacteria while minimizing harm to the host cell. This makes them a compelling option for reducing or substituting antibiotics in semen extenders [27].

The objective of this study was to investigate the antimicrobial peptide properties of BiF2_5K7K against bacteria isolated from sow vaginal discharge and boar semen. Furthermore, we tested the bacterial inhibition efficiency of the BiF2_5K7K antimicrobial peptide when mixed with boar semen extender to investigate its potential as an antibiotic replacement and its effect on extended boar semen quality.

## 2. Results

### 2.1. Minimum Inhibitory Concentration (MIC) and Minimum Bactericidal Concentration (MBC) Assay

The results of MIC and MBC assays of BiF2_5K7K against pathogens isolated from boar semen and sow vaginal discharge are presented in Table 1. Except for *Klebsiella pneumoniae*, *Morganella morganii*, *Proteus mirabilis*, and *Providencia rettgeri* (MIC > 250 µg/mL), MIC values of BiF2_5K7K between 15.625 and 250 µg/mL inhibited the growth of bacteria isolated from boar semen and sow vaginal discharge (Table 1). Meanwhile, MBC values of BiF2_5K7K between 15.625–250 µg/mL showed bactericidal effects. However, several bacteria, including *Klebsiella pneumoniae*, *Morganella morganii*, *Proteus mirabilis*, *Providencia rettgeri*, and *Staphylococcus hyicus*, showed MBC values of more than 250 µg/mL (Table 1).

### 2.2. Total Bacterial Count

The mean total bacterial count of fresh boar semen was log2.27 ± 0.80 CFU/mL (ranged from log1.81 to log2.98 CFU/mL) (Table 2). After incubation at 18 °C, semen samples with different concentrations of BiF2_5K7K were measured at 0, 24, 36, 48, and 72 h, and the results of the total bacterial count are presented in Table 3. With increasing incubation time, the total bacterial count increased. At 0 h after incubation, the lowest total bacterial count was found in the positive control group (log1.22 CFU/mL, BTS plus antibiotic) when compared with other groups. Comparing treatment groups (BTS without antibiotic plus BiF2_5K7K), the total bacterial count varied from log1.33 to log1.47 CFU/mL, which was lower than in the negative control group (log1.79 CFU/mL, BTS without antibiotic). At 24 h after incubation, the pattern of total bacterial count in all groups was more or less the same compared with the slightly increased bacterial count at 0 h. The lowest and highest total bacterial counts were found in the positive control group (log0.67 CFU/mL) and the negative control group (log2.35 CFU/mL), respectively. Meanwhile, the total bacterial count in the treatment groups depended on the concentrations of BiF2_5K7K and varied from log1.51 to log1.84 CFU/mL. The total bacterial count of BiF2_5K7K at concentrations of 62.5 and 31.25 µg/mL at 24 h after incubation were the lowest in the treatment group, and were not significantly different from the positive control group (BTS with antibiotic) (Table 3). The total bacterial count in treatment groups decreased with an increased concentration of BiF2_5K7K (Table 3).

### 2.3. Sperm Quality Parameter Analysis

The sperm quality of fresh boar semen samples is presented in Table 2. On day 1, the sperm quality parameters remained normal and there were no significant differences in all sperm parameters among the six groups, except in the STR (straightness) and LIN (linearity) parameters. The straightness and linearity values of the 15.625 µg/mL group were significantly different from the other groups (75.2% and 33.3%) (Table 4). On day 3, inferior progressive motility occurred in the negative control, 125 µg/mL, and 62.5 µg/mL groups compared with the other groups. The 125 µg/mL dose of BiF2_5K7K had significantly lower effects on sperm motility patterns, including the VCL, VSL, VAP, and ALH parameters, compared with the other groups, especially the negative control group (Table 5). In addition, lower percentages of total motility, viability, intact acrosome, and sperm with high MMP were found in the 125 µg/mL group compared with the other control and treatment groups (Table 5), and these decreased as incubation times increased in some parameters on day 5 (Table 6). On day 5, significantly superior percentages of total motility and progressive motility were found in the 15.625 and 31.25 µg/mL groups compared with the 125 µg/mL group (*p* < 0.05, Table 6). There were significantly lower percentages of sperm motility patterns in the 125 µg/mL group compared with the other groups (*p* < 0.05), except in the STR and LIN parameters. However, there were no significant differences in sperm viability or intact acrosomes among the six groups (Table 6).

### 2.4. Scanning Electron Microscopy

**Figure 1:**
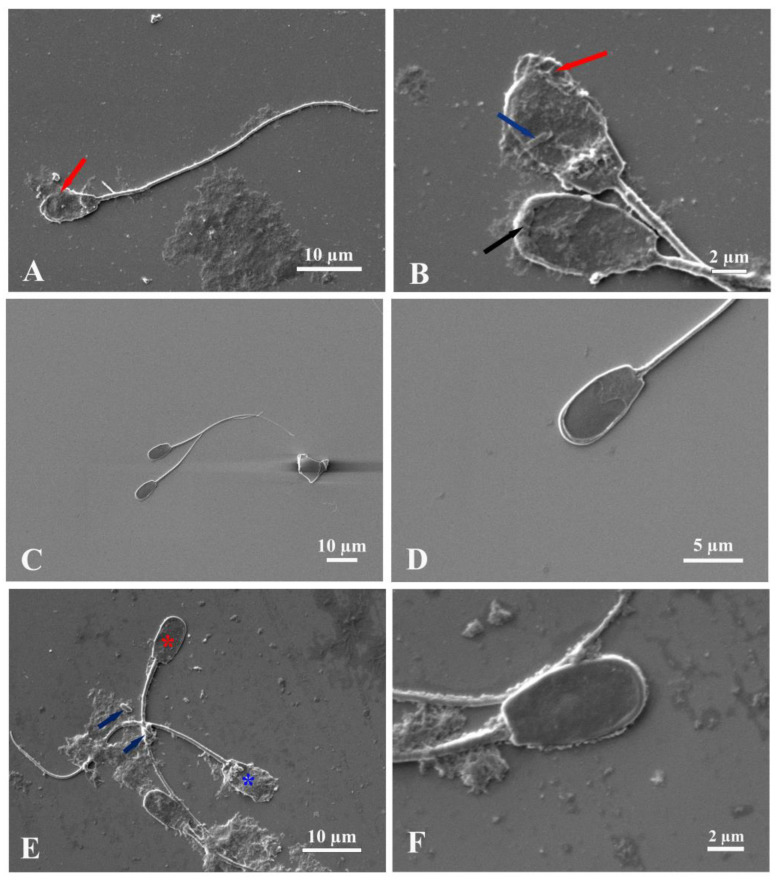
Scanning electron micrographs of boar sperm on day 3 after incubation with non-antibiotic BTS (**A**,**B**), BTS with antibiotic (**C**,**D**), and BiF2_5K7K at 62.5 µg/mL (**E**,**F**). The non-antibiotic BTS group (**A**,**B**) presented sperm with abnormal heads (red arrows), swelling acrosomes (black arrow), and rod bacteria (blue arrows). The BTS with antibiotic group (**C**,**D**) presented normal boar sperm. The BiF2_5K7K at 62.5 µg/mL group (**E**,**F**) showed both normal (red star) and abnormal boar sperm (blue star), and attracted bacteria (**E**) (blue arrow) as well as normal boar sperm at high magnification (**F**).

The sperm morphology evaluation, conducted using scanning electron microscopy (SEM), is presented in Figure 1. The sperm morphology in the positive control group (BTS with antibiotic) revealed normal morphology (Figure 1C,D), whereas abnormal sperm morphology, including membrane detachment, acrosomal damage, and sperm agglutination, as well as the attachment of bacteria on spermatozoa, was found in the negative control group (BTS without antibiotic) (Figure 1A,B). For the 62.5 µg/mL treatment groups (BTS plus BiF2_5K7K at different concentrations), the sperm morphology in these groups showed both normal and abnormal morphology (Figure 1E,F), with a lesser degree of abnormal morphology than in the negative control group (Figure 1E,F).

### 2.5. Fertility Test on the Pig Farm

For the fertility tests on the pig farm, the pregnancy rate, the percentage of pregnancy, the percentage of farrowing rate, the total number of piglets born, the number of piglets born alive, stillbirths, and mummies, as well as the litter birthweight, are presented in Table 7. In the treatment group, BTS supplemented with BiF2_5K7K peptide at a concentration of 31.25 μg/mL showed higher fertility results, such as pregnancy rate, farrowing rate, total number of piglets born, and number of piglets born alive, than those of commercial boar semen extenders.

## 3. Discussion

The results of this study clearly show that BiF2_5K7K inhibits the growth of both Gram-negative and Gram-positive bacteria isolated from boar semen and in extended boar semen. Most of the bacteria contaminating fresh boar semen were *E*. *coli*, *Pseudomonas aeruginosa*, *Proteus mirabilis*, and *Staphylococcus* spp., which is in agreement with the most common bacteria contaminated in fresh boar semen reported by previous studies [8,28,29,30]. In contrast to Ngo et al. [31], Gram-positive bacteria such as *Staphylococcus* spp. were the most frequently contaminated in fresh boar semen. It has been documented that contamination with *Pseudomonas aeruginosa* and *E*. *coli* negatively impacts boar sperm through either causing sperm agglutination or decreasing sperm motility [5,29]. The presence of *E. coli* in boar semen prior to artificial insemination is primarily responsible for sow endometritis and accounts for 72.3% of cases [5,32,33]. Clinical endometritis often presents with vaginal discharge, which can be attributed to various factors, including hormonal imbalance [34] or post-ovulatory insemination [32]. While a range of antibiotics can help reduce the severity of acute endometritis, it is important to note that this condition can worsen and develop into chronic endometritis, which can have a significant negative effect on pigs’ reproductive performance [35]. BiF2_5K7K at concentrations of 15.625 and 31.25 showed an ability to inhibit bacteria isolated from boar semen for only 24 h; however, it did not show a deleterious effect on semen quality during storage for 3 days. As a result, this peptide might be an alternative to antibiotics for supplementation into boar semen extenders in order to diminish the negative effect of bacterial contamination in fresh boar semen. In commercial semen extenders, antibiotics including amoxicillin, gentamicin, neomycin, penicillin, and streptomycin are added and widely used to inhibit the growth of bacteria [12,13,29,36]. These antibiotics have also been routinely used for the treatment of bacterial infections via both injection and feed medication on many pig farms worldwide. It has been reported that bacteria isolated from boar semen [8] and diarrheic piglets [10] show high resistance to many antibiotics. This is in accordance with the present result, where we found that some bacteria (i.e., *Enterobacter* spp., *Klebsiella* spp., *Proteus* spp., *Providencia* spp., and *Staphylococcus* spp.) had high levels of MIC and MBC. It is worth noting that at concentrations of 250 µg/mL, BiF2_5K7K cannot inhibit *Proteus mirabilis*, whilst it can inhibit *E*. *coli* and *Pseudomonas aeruginosa* at concentrations of 15.625 and 31.25 µg/mL, respectively. Similar outcomes were also reported when using synthetic cyclic hexapeptides c-WWW and c-WFW, which are unable to inhibit *Proteus* spp. [9,37]. Considering the results of the total bacterial count of fresh boar semen on day 0 and extended boar semen from days 1 to 5 in all groups, the BiF2_5K7K antimicrobial peptide at concentrations of 15.625, 31.25, 62.5, and 125 µg/mL is able to inhibit bacterial growth in extended boar semen stored at 18 °C for at least 24 h after incubation. The number of bacterial counts in those treatment groups was as low as log1.51 to log1.78 CFU/mL, respectively, when compared with the bacterial count of log2.35 CFU/mL in the negative control group. It has been reported that the total bacteria count in fresh boar semen should range from 22.40 to 188.20 (×10^3^ CFU/mL) in order to optimize reproduction in pig farms [38]; moreover, a reduction in sperm viability of 6.4% has been documented, which corresponds to every log10 increase in the total bacterial count [31]. Previous research has indicated that the quality of boar semen is affected by the bacteria count. It has been reported that sperm viability decreases with an *E. coli* concentration of approximately 10^3^ CFU/mL [39]. In addition, boar semen contaminated with an *E. coli* concentration greater than 3.5 × 10^3^ CFU/mL resulted in inferior reproductive performance by reducing litter size in pig farms [35]. Fertilizing ability was also found to have decreased by 10^4^–10^6^ CFU/mL in boar semen contaminated with *Pseudomonas aeruginosa* [40]. Moreover, in a study of the effects of anaerobic bacteria, including *Clostridium perfringens*, the total motility of boar semen was reduced at a concentration of 10^7^–10^8^ CFU/mL [41]. Although, after storage for 36 h, the total bacterial count in treatment groups increased from log1.51 to log1.78 at 24 h to log0.85 to log3.82 CFU/mL, this total bacterial count was considerably lower than in the negative control group (log3.47 CFU/mL, BTS without antibiotic). This indicates that pig farmers can use the alternative BiF2_5K7K peptide as a replacement for antibiotics in semen extenders; however, this extended semen should be used for artificial insemination within 24 h and no later than 36 h in order to avoid deleterious effects from a high number of bacteria.

Considering semen quality from days 0 to 5 in different groups, despite the fact that the total bacterial count increased over time after storage of the extended boar semen at 18 °C, this negative effect on semen quality was not observed until day 3; in particular, a negative effect was observed in progressive motility values in a BiF2_5K7K concentration of 125 µg/mL. Only BiF2_5K7K at concentrations of 31.25 and 15.625 µg/mL was able to maintain all sperm parameters comparable to the positive control group (BTS with antibiotic). On day 5, a BiF2_5K7K concentration of 125 µg/mL showed lesser total motility, progressive motility, and mitochondrial membrane potential than other groups. This might be due to the fact that too high a concentration of antimicrobial peptide may cause deleterious effects on the spermatozoa [19]. During storage on days 3 and 5, besides the negative effect found for sperm parameters, as mentioned above, the sperm morphology determined via scanning electron microscopy revealed that most of the plasma membrane damage around the head and acrosome region was found in the BTS without an antibiotic group, which may have been caused by a high number of bacteria, as described earlier by Bonet et al. [42]. It is worth noting that the ability to inhibit bacterial growth without damaging spermatozoa is considered an imperative property of the antimicrobial peptide for supplementation in boar semen extenders [9,19,43]. It has been suggested that direct and rapid binding to the external bacterial cell wall, such as lipopolysaccharide (LPS) in Gram-negative bacteria or teichoic acid in Gram-positive bacteria, might be the mechanism through which antimicrobial peptides interact [22,44,45], due to the fact that there is a difference in charge between the bacterial and animal membranes. Although the total net charge of the sperm is negative on the sperm head, the sperm head position has a positive charge [46]. Consequently, the possibility of interacting with AMPs is lower; as a result, antimicrobial peptides act on the bacterial membrane rather than the sperm membrane [41,47]. The positive charge AMPs have strong interactions with the negative charge on the outermost bacterial cell surface due to the presence of lipopolysaccharides or teichoic acid [45,47,48,49]; however, the AMPs have weak interactions with the sperm membrane, which has a negative charge in the innermost region near the cytoplasm [11,44]. It has previously been reported that certain antimicrobial peptides such as Nisin and Protegrine 1 (PG 1) have a detrimental effect on sperm [50,51]. Nisin has shown a prompt effect on spermicidal activities and immobilization of spermatozoa within 20 s; moreover, the toxic dose of Nisin varies from 50–400 µg depending on the animal species [50]. PG 1 has also shown a negative effect on sperm motility and viability, although it has demonstrated compromised antibacterial activity when compared with the antibiotic group [51]. Even though antimicrobial peptides have shown greater antimicrobial activity at high concentrations than at low concentrations, they can also damage boar spermatozoa during storage at 17 °C, as reported by Shaoyong et al. [52]. A combination of antimicrobial peptides and antibiotics is occasionally used for reducing antibiotics and reducing the negative effect of too high a concentration of antimicrobial peptide on boar sperm. It has been shown that a combination of 0.16 g/L of epsilon-polylysine (ε-PL) and 0.125 g/L of gentamycin provides sperm quality equivalent to adding 0.25 g/L of gentamycin alone in liquid-stored pig semen [52]. Nevertheless, utilizing BiF2_5K7K as a semen extender at a concentration of 31.25 μg/mL may lead to increased production expenses. Hence, further investigation may be necessary to diminish the concentration of BiF2_5K7K, such as by combining minimal amounts of antibiotics with BiF2_5K7K or using two or more AMPs as a cocktail peptide. Moreover, the use of a single antimicrobial peptide, an antimicrobial peptide combined with a commercial antibiotic, or a combination of antimicrobial peptides in order to cope with multidrug-resistant bacteria has also been reported [53]. The short-term semen extender (BTS) utilized in the present study can preserve semen quality for a maximum of three days after dilution [54]. However, it may be stored for as long as five days [55]. For the reasons mentioned above, this study evaluated the sperm quality at days 0, 1, 3, and 5 after storage to ensure sure the BTS maintained the sperm quality ensured by the manufacturer. The total bacterial count was determined after 0, 24, 36, 48, and 72 h of storage, as the bacteria increased rapidly during storage and significantly after 72 h [2,5,56]. It is also important to emphasize that extended boar semen was generally utilized by the pig farms within 24 h of storage. Consequently, the present experimental design corresponded to standard clinical practice.

In the present study, the antimicrobial peptide BiF2_5K7K showed its effectiveness against both Gram-positive and Gram-negative bacteria isolated from fresh boar semen and sow vaginal discharge. This antimicrobial peptide not only has an effect on antibacterial activity, but also causes less damage to boar sperm during storage at 17 °C compared with antibiotics. In this study, the toxic impact of BiF2_5K7K was observed at high concentrations (62.5 and 125 µg/mL) and varied based on the incubation time during storage in boar sperm. Considering the sperm quality and the total bacterial count in each treatment group, it seems likely that BiF2_5K7K at concentrations of 15.625 and 31.25 µg/mL are the optimal doses to replace antibiotics in boar semen extenders. For the fertility test, it has been demonstrated in bovine sperm that beta-defensin 126 improves sperm motility but does not promote the fertilizing ability during in vitro tests [57]. BiF2_5K7K at a concentration of 31.25 µg/mL was selected to test the impact on reproductive performance in relation to the standard farm condition (BTS with antibiotic) because of the bacterial inhibitory effect after incubation at 24 h (log1.51 ± 0.29 CFU/mL) and the less negative effect on semen quality. The reproductive performance parameter for the above experiment was based on the work of Koketsu et al. [58]. In agreement with the present results, the fertility test conducted on a pig farm using BiF2_5K7K supplemented in boar semen extenders for artificial insemination showed a superior pregnancy rate, farrowing rate, total number of piglets born, and number of piglets born alive compared with the commercial semen extender used at the pig farm. BiF2_5K7K has the ability to inhibit bacterial growth without affecting the efficiency of reproductive ability, which is no different from the use of BTS with antibiotics. The extended boar semen in the present study, which contained BiF2_5K7K, may be responsible for the positive effects observed on various reproductive parameters, including the increase in the number of piglets born alive, the improvement in the farrowing rate and pregnancy rate, and other related factors. Antimicrobial peptides, such as β-defensin, cathelicidin, PMAP23, and PMAT37, have been observed to be present in the endometrium of female pigs during the reproductive cycle and in the placental tissue of pregnant sows [59,60]. The mechanism underlying of the particular antimicrobial peptides could influence the number of piglets born need further studies. Therefore, BiF2_5K7K may be of interest to the swine industry in order to minimize the use of antibiotics in pig farms. Nevertheless, it is worth noted that field fertility test was performed by using 20 sows in each group, and as a result, a further experiment with a greater number of sows in each group may be needed.

## 4. Materials and Methods

### 4.1. Peptide Synthesis

The BiF2_5K7K antimicrobial peptide was inspired by natural AMPs, as previously described [61,62]. According to the 2020 study by Klubthawee et al. [62], peptide synthesis methods were used. As trifluoroacetate salts, the BiF2_5K7K components were purified using HPLC after being produced using solid-phase methods and 9-fluorenylmethoxycarbonyl (Fmoc) chemistry (ChinaPeptides, Shanghai, China). The content of residual TFA, quantified using 19F nuclear magnetic resonance (NMR), was less than 1.7% (wt/wt). Dehydration condensation was used for producing the TAMRA-labeled BiF2_5K7K, and an amide bond at the N-terminus was utilized for attaching TAMRA to BiF2_5K7K. Analytical reversed-phase HPLC determined that all of the peptides were more than 98% purified. Electrospray ionization mass spectrometry (ESI-MS) was used to identify the peptides.

The characteristics of BiF2_5K7K are presented in Table 8. BiF2_5K7K consisted of 12 amino acid sequence peptides with a molecular weight of 1541.07 g/mol. The net electric charge and hydrophobicity were +6 and 0.336, respectively [61].

### 4.2. Minimum Inhibitory Concentration (MIC) and Minimum Bactericidal Concentration (MBC) Test

The MIC assay of BiF2_5K7K was conducted using 12 bacteria isolated from boar semen and sow vaginal samples from the stock collection of the Bacterial Laboratory, Veterinary Diagnostic Center, Faculty of Veterinary Science, Mahidol University, Thailand. The bacterial stock was kept in glycerol at −80 °C. Bacteria from the culture stock were cultured on MacConkey agar (Difco, Reno, NV, USA) or sheep blood agar (Biomedia, Nonthaburi, Thailand), and then incubated for 18 to 24 h at 37 °C. After that, one to three bacterial colonies were transferred into a regular saline solution (0.85% NaCl) and thoroughly mixed. The turbidity of the bacterial sample was measured using a 0.5 McFarland standard, approximately 10^8^ CFU/mL. Following guidelines from the Clinical and Laboratory Standards Institute (CLSI), the broth microdilution method was used to conduct the MIC assay. The assays were performed in triplicate using 96-well plates. In each well, 100 μL of the bacterial suspension, which had been diluted in Mueller–Hinton broth (Difco, USA) to 10^6^ CFU/mL, was added to 100 μL of the appropriate dilutions of BiF2_5K7K at the selected concentrations using a two-fold dilution method (1.953–250 µg). As a control, a medium without BiF2_5K7K was used. After incubation, the MIC values were determined and defined as the lowest concentration of each BiF2_5K7K at which evidence of bacterial growth was absent. After the MIC assay, 100 µL aliquots from each well of bacterial growth representing the MIC values were streaked on the culture media agar plate and incubated for 18–24 h at 37 °C. The MBC value was defined as the lowest concentration of BiF2_5K7K at which nonbacterial colonies did not proliferate on the culture media agar.

### 4.3. Boar Semen Collection and Preparation

Ten individual adult boars of ages ranging from 1.5 to 3 years were chosen for semen collection. The gloved-hand method was used to collect sperm from each boar. Semen was filtered through gauze, and only sperm-rich fractions were collected during the collection process. The sperm motility, concentration, percentage of viability, intact acrosomes, mitochondrial membrane potential, osmolality, and total bacterial concentration of the fresh semen were measured after collection. Only semen ejaculates with progressive motility values of more than 70% and concentrations of more than 100 × 10^6^ spermatozoa/mL were included in the experiment.

As shown in Table 9, the fresh boar semen was divided into 6 groups via dilution with Beltsville Thawing Solution (BTS; Minitube, Tiefenbach, Germany), BTS with antibiotic (Minitube, Tiefenbach, Germany), and BTS without antibiotic plus various concentrations of BiF2_5K7K. Each group’s sperm concentration was 4.5 × 10^9^ spermatozoa/100 mL. The diluted semen samples were incubated at 18 °C until evaluation. After incubation, the total bacterial concentration was assessed at 0, 24, 36, 48, and 72 h. The quality of sperm was evaluated on days 1, 3, and 5 after storage.

### 4.4. Total Bacterial Count

The spread plate technique was employed to ascertain the total bacterial *count* subsequent to the incubation of a boar semen sample at 18 °C. The semen samples were subjected to ten-fold dilution with normal saline solution (0.85% NaCl). One hundred microliters (µL) of each semen sample dilution were evenly distributed on Plate Count Agar (PCA) (Difco, Nevada, USA) and incubated at 37 °C. After 48 h of incubation, the colonies were enumerated and converted into colony-forming units per milliliter (CFU/mL).

### 4.5. Sperm Parameter Analysis

#### 4.5.1. Sperm Motility

Computer-assisted sperm motility analysis (CASA) was used to examine sperm motility (AndroVision^®^, Minitube, Tiefenbach, Germany). In brief, 3 µL of extended semen was pipetted into a pre-warmed counting chamber (Leja^®^, IMV Technologies, L’Aigle, Basse-Normandie, France) and then immediately measured using CASA software (REF.: 12500/0000). Five fields of each sample were evaluated, and at least 600 cells were counted per analysis. The analysis results expressed the percentages of motile sperm and progressive motile sperm, as well as motility patterns including curvilinear velocity (VCL, µm/s), average pathway velocity (VAP, mm/s), straight-line velocity (VSL, mm/s), amplitude of lateral head displacement (ALH, mm), straightness (STR; VSL/VAP, %), and linearity (LIN; VSL/VCL, %) [63,64].

#### 4.5.2. Sperm Viability

The viability of the sperm was examined using Ethidium homodimer-1 (EthD-1, E1169, Invitrogen, Waltham, MA, USA) and SYBR-14 (Sperm viability kit, Molecular probes, L7011, Thermo Fisher Scientific, Waltham, MA, USA). SYBR-14 (0.54 µM in DMSO) and EthD-1 (1.17 µM in PBS) were combined with an aliquot of 10 μL of the semen samples and then incubated at 37 °C for 15 min. After incubation, 5 µL of the processed sample was placed onto a pre-warmed glass slide and covered with a coverslip. A total of 200 sperm were assessed under a fluorescence microscope at 1000× magnification and classified as live or dead sperm [63,64].

#### 4.5.3. Sperm Acrosomal Integrity

The acrosomal integrity of the sperm was evaluated by using fluorescein isothiocyanate-labeled peanut (*Arachis hypogaea*) agglutinin (FITC-PNA) with EthD-1 staining. Next, 10 µL samples of the diluted semen were mixed with 10 µL of EthD-1 and incubated at 37 °C for 15 min. Five µL of the mixture was smeared on a glass slide and fixed with 95% ethanol for 30 s. Each glass slide was covered with 50 µL of FITC-PNA (diluted with PBS 1:10 *v*/*v*) and incubated in a moist chamber at 4 °C for 30 min. After incubation, each sample was rinsed with cold PBS and air-dried. A total of 200 sperm were assessed using a fluorescent microscope and classified as intact acrosomes or damaged acrosomes [54,63,65].

#### 4.5.4. Sperm with High Mitochondrial Membrane Potential (MMP)

Fluorochrome 5,5′,6,6′-tetrachloro-1,1′,3,3′-tetraethylbenzimidazolyl-carbocyanine iodide (1.53 mM) (JC-1; T3168, Invitrogen, Waltham, MA USA) was used in the staining process to determine the mitochondrial membrane potential of the sperm. A sample of 50 µL of diluted semen was mixed with 3 µL of a 1.53 mM JC-1 solution and 3 µL of a 2.4 mM propidium iodide (PI) solution in DMSO. The mixture was then incubated for 10 min at 37 °C in a dark container. Two hundred sperm were analyzed and divided into groups according to their degree of mitochondrial membrane potential using a 400× magnification fluorescent microscope [63,66].

### 4.6. Scanning Electron Microscopy (SEM)

Sperm samples were subjected to evaluation for morphology under a scanning electron microscope by using the classical conventional procedure as follows: the semen samples were fixed with 2.5% glutaraldehyde (Electron Microscopy Sciences, Hatfield, UK) in PBS for 24 h. After fixation, a washing process with PBS was conducted for 15 min and repeated three times. The samples were then stained with 0.1% osmium tetroxide (Sigma-Aldrich, Darmstadt, Germany) for 1 h and washed three times for 15 min with PBS. In the dehydration step, the samples were dehydrated with a graded series of ethanol at concentrations of 70%, 80%, 90%, and 95% absolute ethanol. The semen samples were processed and then placed onto an SEM stub and coated with 50 nm platinum particles [42]. Finally, the sperm morphology was observed under the scanning electron microscope (JEOL, JSM-IT500LA, Tokyo, Japan).

### 4.7. Fertility Test on the Pig Farm

After weaning, estrus was detected twice a day by monitoring the vulva for swelling and redness, as well as by performing a back-pressure test when a boar was around [54]. All the sows were inseminated thrice with a conventional AI catheter at 12 h, 24 h, and 36 h after standing estrus with a dose of semen (boar of proven fertility). The semen dose contained 3 × 10^9^ spermatozoa in 80 mL of BTS with gentamicin (control, *n* = 20) and BTS supplemented with 31.25 μg/mL of BiF2_5K7K peptide (treatment, *n* = 20), and was stored at 18 °C for no more than 24 h. The pregnancy tests were performed on days 23–24 of pregnancy via transabdominal ultrasonography, real-time B-mode (50STringa, sector probe with 5 MHz, ESAOTE Pie Medical, Maastricht, The Netherlands) [67]. The pregnancy rate; the percentage of farrowing rate; the total number of piglets born; and the number of piglets born alive, dead, and mummified, as well as litter birthweight, were recorded.

### 4.8. Statistical Analysis

For the MIC and MBC data, the descriptive statistic was applied. Using PASW Statistics for Windows, version 18.0 (SPSS Inc., Chicago, IL, USA), the statistical analysis was performed. The Shapiro–Wilk test was used to evaluate the data distribution, and the results showed a normal distribution (*p* > 0.05). The total bacteria count and the fertility data were presented as mean ± SD. The semen parameter data included total motility, progressive motility, curvilinear velocity, straight-line velocity, average pathway velocity, amplitude of lateral head displacement, straightness, and linearity, as well as sperm with high mitochondrial membrane potential, and were presented as mean ± SEM. The bacterial count and sperm parameter data analysis were performed using the one-way analysis of variance (ANOVA) test, and mean values were compared using Duncan’s test. Data on fertility were compared using the Student’s t-test, and the Chi-square test was used for the pregnancy and farrowing rates. When dealing with non-normally distributed data, the Mann–Whitney U (Wilcoxon’s rank sum) test was applied. Statistical significance was determined at a *p*-value < 0.05.

## 5. Conclusions

In this study, the BiF2_5K7K antimicrobial peptide demonstrated the ability to inhibit the growth of bacteria isolated from boar semen, and, thus, appears to be a worthy alternative to antibiotics in boar semen extenders. Nevertheless, the successful application of this particular AMP depends on the concentration and incubation time during storage. According to the present study’s findings, adding BiF2_5K7K at a concentration of 31.52 µg/mL in a BTS semen extender without antibiotics, with storage at 18 °C for 24 h, demonstrated the most effective bacterial-inhibitory effect. Furthermore, the 31.52 µg/mL BiF2_5K7K concentration demonstrated the least harmful impact on boar semen parameters and revealed superior fertility when tested on a pig farm.

## Figures and Tables

**Table 1:** MIC and MBC values of BiF2_5K7K tested against 12 bacteria isolated from boar semen and sow vaginal discharge.

Gram	ID	Sample	Bacteria	BiF2_5K7K	
				MIC (µg/mL)	MBC (µg/mL)
Negative	S1LLF	Boar semen	*Citrobacter koseri*	15.625	31.25
	S8-6LF	Boar semen	*Enterobacter hormaechei*	250	250
	S5LF3	Boar semen	*Escherichia coli*	15.625	15.625
	MI912-2LF/62	Sow vaginal discharge	*Klebsiella pneumoniae*	>250	>250
	V5-6	Sow vaginal discharge	*Morganella morganii*	>250	>250
	S6-4	Boar semen	*Providencia alcalifaciens*	15.625	62.5
	S3	Boar semen	*Proteus mirabilis*	>250	>250
	V2-5	Sow vaginal discharge	*Providencia rettgeri*	>250	>250
	S2NLF	Boar semen	*Pseudomonas aeruginosa*	31.25	125
	V4-3	Sow vaginal discharge	*Pasteurella aerogenes*	125	125
Positive	S7-5W	Boar semen	*Staphylococcus sciuri*	15.625	15.625
	V2-3	Sow vaginal discharge	*Staphylococcus hyicus*	125	>250

**Table 2:** Descriptive statistics for sperm parameter measurements of fresh boar semen (*n* = 10).

Semen Parameters	Mean ± S.D.	Range
Concentration (×10^6^ spz/mL)	211.50 ± 71.10	146–345
Osmolality (mOsm/kg)	304.80 ± 8.50	288–315
Total motility (%)	94.40 ± 3.90	86.60–99.40
Progressive motility (%)	90.90 ± 5.80	80.00–98.50
Sperm viability (%)	88.3 0± 2.80	85–93
Intact acrosome (%)	85.40 ± 2.90	80–91
MMP (%)	82.50 ± 2.70	80–89
Total bacterial count (CFU/mL)	log1.81 ± 0.80	log1.81–log2.98

MMP: Sperm with high mitochondrial membrane potential.

**Table 3:** Total bacteria count (mean ± SEM) from boar semen samples (*n* = 10) at 0, 24, 36, 48, and 72 h after incubation at 18 °C.

Group	Concentration (µg/mL)	Total Bacterial Count (log) (CFU/mL)				
		Incubation Time				
		0 h	24 h	36 h	48 h	72 h
BTS	-	1.79 ± 0.23	2.35 ± 0.26 ^b^	3.47 ± 0.58 ^b^	3.98 ± 0.76 ^b^	6.25 ± 1.75 ^b^
BTS + ABO	-	1.22 ± 0.52	0.67 ± 0.33 ^a^	0.85 ± 0.15 ^a^	1.12 ± 0.42 ^a^	0.00 ± 0.00 ^a^
BiF2_5K7K *	125	1.33 ± 0.30	1.78 ± 0.31 ^b^	2.58 ± 1.06 ^b^	3.55 ± 0.97 ^b^	6.14 ± 2.79 ^b^
BiF2_5K7K *	62.5	1.42 ± 0.34	1.53 ± 0.31 ^a^	3.36 ± 0.75 ^b^	3.72 ± 0.83 ^b^	6.23 ± 2.68 ^b^
BiF2_5K7K *	31.25	1.39 ± 0.27	1.51 ± 0.29 ^a^	3.21 ± 0.89 ^b^	4.32 ± 1.15 ^b^	6.59 ± 2.82 ^b^
BiF2_5K7K *	15.625	1.47 ± 0.19	1.84 ± 0.33 ^b^	3.82 ± 0.94 ^b^	3.02 ± 1.03 ^b^	7.09 ± 2.16 ^b^

* Combination of BiF2_5K7K and BTS without antibiotics. ^a,b^ Significant difference among groups at the same incubation time (*p* < 0.05). ABO: antibiotic; BTS: Beltsville Thawing Solution.

**Table 4:** Mean ± SEM of semen quality parameters on day 1 after incubation at 18 °C (*n* = 10).

Sperm Parameters	Group					
	BTS	BTS + ABO	BiF2_5K7K 125 µg/mL *	BiF2_5K7K 62.50 µg/mL *	BiF2_5K7K 31.25 µg/mL *	BiF2_5K7K 15.625 µg/mL *
MOT (%)	90.5 ± 1.6	90.7 ± 1.5	90.3 ± 1.3	92.3 ± 1.0	92.6 ± 1.0	90.5 ± 1.1
PMOT (%)	81.8 ± 2.6	81.23 ± 2.8	81.3 ± 2.0	84.1 ± 2.0	83.8 ± 1.9	81.6 ± 2.2
VCL (µm/s)	120.4 ± 9.4	125.4 ± 8.2	188.1 ± 6.9	116.1 ± 6.3	113.8 ± 6.9	110.6 ± 5.6
VSL (µm/s)	37.4 ± 3.1	38.8 ± 3.7	37.2 ± 3.4	37.7 ± 3.1	37.3 ± 3.1	36.8 ± 2.4
VAP (µm/s)	51.4 ± 3.6	53.9 ± 3.9	50.9 ± 3.6	50.8 ± 3.3	49.9 ± 3.3	48.6 ± 2.5
ALH (µm)	1.18 ± 0.09	1.21 ± 0.67	1.14 ± 0.05	1.11 ± 0.04	1.08 ± 0.04	1.06 ± 0.04
STR (%)	72.1 ± 1.7 ^a^	71.2 ± 1.8 ^a^	72.2 ± 1.7 ^a^	73.89 ± 1.5 ^a^	74.0 ± 1.3 ^a^	75.2 ± 1.3 ^b^
LIN (%)	31.1 ± 1.2 ^a^	30.7 ± 1.4 ^a^	31.1 ± 1.3 ^a^	32.2 ± 1.1 ^a^	32.6 ± 1.0 ^a^	33.3 ± 0.8 ^b^
Viability (%)	85.4 ± 0.9	86.3 ± 0.8	84.0 ± 0.7	85.5 ± 0.5	86.3 ± 0.8	84.8 ± 0.5
Intact acrosome (%)	83.5 ± 0.8	83.8 ± 0.7	82.4 ± 0.9	82.4 ± 0.8	82.5 ± 1.0	82.7 ± 0.9
MMP (%)	77.2 ± 1.6	78.3 ± 1.2	77.7 ± 1.0	77.9 ± 1.2	79.9 ± 1.4	78.7 ± 0.9

* Combination of BiF2_5K7K and BTS without antibiotics. ^a,b^ Significant difference among groups at the same incubation time (*p* < 0.05). ABO: antibiotic; BTS: Beltsville Thawing Solution; MOT: total motility; PMOT: progressive motility; VCL: curvilinear velocity; VSL: straight-line velocity; VAP: average pathway velocity; ALH: amplitude of lateral head displacement; STR: straightness; LIN: linearity; MMP: sperm with high mitochondrial membrane potential.

**Table 5:** Mean ± SEM of semen quality parameters on day 3 after incubation at 18 °C (*n* = 10).

Sperm Parameters	Group					
	BTS	BTS + ABO	BiF2_5K7K 125 µg/mL *	BiF2_5K7K 62.50 µg/mL *	BiF2_5K7K 31.25 µg/mL *	BiF2_5K7K 15.625 µg/mL *
MOT (%)	85.9 ± 2.7	86.1 ± 2.6	73.3 ± 6.9	85.5 ± 3.4	87.5 ± 2.9	86.3 ± 2.9
PMOT (%)	73.3 ± 3.9 ^a^	73.4 ± 4.1 ^a^	60.8 ± 7.3 ^b^	73.6 ± 4.6 ^a^	76.4 ± 3.9 ^a^	75.1 ± 4.2 ^a^
VCL (µm/s)	106.3 ± 10.9 ^a^	109.9 ± 12.1 ^a^	89.8 ± 13.9 ^b^	107.1 ± 11.9 ^a^	102.2 ± 11.7 ^a,b^	100.4 ± 11.5 ^a,b^
VSL (µm/s)	33.0 ± 3.5 ^a^	34.1 ± 4.1 ^a^	27.8 ± 4.7 ^b^	35.0 ± 4.5 ^a^	33.6 ± 4.2 ^a^	33.4 ± 4.3 ^a^
VAP (µm/s)	45.3 ± 4.7 ^a^	47.4 ± 5.4 ^a^	38.2 ± 5.9 ^b^	46.0 ± 5.5 ^a^	44.3 ± 5.3 ^a,b^	44.0 ± 5.1 ^a,b^
ALH (µm)	1.09 ± 0.09 ^a^	1.09 ± 0.10 ^a^	0.94 ± 0.12 ^b^	1.05 ± 0.09 ^a,b^	1.01 ± 0.09 ^a,b^	0.99 ± 0.09 ^a,b^
STR (%)	72.9 ± 1.0 ^a^	71.8 ± 1.3 ^a^	72.1 ± 1.5 ^a^	75.0 ± 1.4 ^a,b^	75.8 ± 1.7 ^b^	75.4 ± 1.2 ^b^
LIN (%)	31.2 ± 0.0 ^a,b^	31.0 ± 0.9 ^a,b^	30.8 ± 0.8 ^a^	32.7 ± 1.1 ^a,b^	32.9 ± 1.1 ^a,b^	33.1 ± 1.0 ^b^
Viability (%)	81.6 ± 1.5	82.1 ± 1.4	77.6 ± 2.3	80.3 ± 1.7	82.8 ± 1.1	81.3 ± 1.1
Intact acrosome (%)	80.0 ± 1.5	80.3 ± 0.9	75.3 ± 0.3	78.6 ± 1.5	79.4 ± 1.2	78.6 ± 1.2
MMP (%)	71.3 ± 1.8	72.0 ± 2.3	66.4 ± 3.8	72.4 ± 2.3	73.9 ± 2.5	74.1 ± 2.0

* Combination of BiF2_5K7K and BTS without antibiotics. ^a,b^ Significant difference among groups at the same incubation time (*p* < 0.05). ABO: antibiotic; BTS: Beltsville Thawing Solution; MOT: total motility; PMOT: progressive motility; VCL: curvilinear velocity; VSL: straight-line velocity; VAP: average pathway velocity; ALH: amplitude of lateral head displacement; STR: straightness; LIN: linearity; MMP: sperm with high mitochondrial membrane potential.

**Table 6:** Mean ± SEM of semen quality parameters on day 5 after incubation at 18 °C (*n* = 10).

Sperm Parameters	Group					
	BTS	BTS + ABO	BiF2_5K7K 125 µg/mL *	BiF2_5K7K 62.50 µg/mL *	BiF2_5K7K 31.25 µg/mL *	BiF2_5K7K 15.625 µg/mL *
MOT (%)	72.6 ± 7.2 ^a,b^	79.3 ± 4.7 ^a^	57.3 ± 10.1 ^b^	75.5 ± 6.5 ^a,b^	80.0 ± 5.6 ^a^	80.3 ± 4.8 ^a^
PMOT (%)	60.2 ± 7.6 ^a,b^	66.0 ± 5.3 ^a,b^	45.9 ± 10.1 ^b^	63.9 ± 7.7 ^a,b^	69.8 ± 6.5 ^a^	71.1 ± 6.0 ^a^
VCL (µm/s)	79.7 ± 11.8 ^a^	94.9 ± 10.7 ^a^	71.2 ± 16.3 ^b^	90.6 ± 14.1 ^b^	95.2 ± 14.4 ^b^	94.4 ± 12.5 ^b^
VSL (µm/s)	24.4 ± 3.9 ^a^	29.9 ± 3.7 ^b^	23.5 ± 6.1 ^a^	28.1 ± 5.2 ^a,b^	30.3 ± 5.5 ^a,b^	29.8 ± 4.9 ^a,b^
VAP (µm/s)	33.6 ± 5.1 ^a^	41.0 ± 4.7 ^b^	30.9 ± 7.4 ^c^	38.0 ± 6.4 ^a,b^	40.7 ± 6.8 ^a,b^	39.6 ± 5.9 ^a,b^
ALH (µm)	0.86 ± 0.11 ^a^	0.98 ± 0.09 ^a^	0.76 ± 0.13 ^b^	0.93 ± 0.12 ^a^	0.96 ± 0.11 ^a^	0.95 ± 0.09 ^a^
STR (%)	71.4 ± 1.3	72.4 ± 1.7	72.9 ± 2.0	72.8 ± 1.3	73.0 ± 1.5	73.6 ± 1.6
LIN (%)	30.0 ± 0.6	31.4 ± 1.1	31.1 ± 1.3	30.0 ± 1.1	30.8 ± 1.5	30.4 ± 1.3
Viability (%)	79.2 ± 2.0	79.6 ± 1.0	73.1 ± 3.3	78.9 ± 1.2	79.6 ± 1.8	79.6 ± 1.4
Intact acrosome (%)	74.8 ± 1.4	77.1 ± 0.9	73.0 ± 3.0	77.1 ± 0.8	77.8 ± 1.5	78.1 ± 1.3
MMP (%)	64.4 ± 2.5 ^a^	66.0 ± 2.7 ^a^	51.0 ± 6.7 ^b^	64.2 ± 3.5 ^a^	64.6 ± 3.0 ^a^	67.9 ± 1.7 ^a^

* Combination of BiF2_5K7K and BTS without antibiotics. ^a,b,c^ Significant difference among groups at the same incubation time (*p* < 0.05). ABO: antibiotic; BTS: Beltsville Thawing Solution; MOT: total motility; PMOT: progressive motility; VCL: curvilinear velocity; VSL: straight-line velocity; VAP: average pathway velocity; ALH: amplitude of lateral head displacement; STR: straightness; LIN: linearity; MMP: sperm with high mitochondrial membrane potential.

**Table 7:** Reproductive performance (mean ± SD) of sows inseminated using liquid stored semen with commercial BTS with antibiotic (control) and BTS supplemented with 31.25 µg/mL of BiF2_5K7K peptide (treatment).

Parameters	Groups	
	Control (*n* = 20)	Treatment (*n* = 20)
Average parity	3.7 ± 0.4	3.7 ± 0.5
Pregnancy rate (%)	90.0 ± 0.3	100.0 ± 0.0
Farrowing rate (%)	80.0 ± 0.4	85.0 ± 0.4
Total number of piglets born	12.6 ± 3.0	14.1 ± 2.6
Number of piglets born alive	10.8 ± 3.1 ^a^	13.1 ± 2.4 ^b^
Stillborn piglets (%)	0.06 ± 0.25	0.17 ± 0.52
Mummified fetuses (%)	0.0 ± 0.0	0.0 ± 0.0
Litter birthweight (kg)	15.5 ± 4.9 ^a^	18.8 ± 3.9 ^b^

^a,b^ Significant difference using Student’s *t*-test between control and treatment groups (*p* < 0.05).

**Table 8:** Physicochemical properties of the BiF2_5K7K peptide.

Peptide	Amino acid Sequence	Number of Amino Acids	Molecular Weight (g/mol)	Net Charge	Hydrophobicity	Percentage of Hydrophobic Residues
BiF2_5K7K	FLVKKIKKILRR	12	1541.07	+6	0.336	50%

**Table 9:** Group of experiments with varying BiF2_5K7K concentrations.

Group	Antimicrobial Peptide	Concentration (µg/mL)
Group 1	Negative control (BTS)	-
Group 2	Positive control (BTS with gentamicin)	-
Group 3	BiF2_5K7K *	125
Group 4	BiF2_5K7K *	62.50
Group 5	BiF2_5K7K *	31.25
Group 6	BiF2_5K7K *	15.625

* Combination of BiF2_5K7K and BTS without antibiotics.

## Data Availability

The original contributions presented in the study are included in the article; further inquiries can be directed to the corresponding author.
